# Mid-Infrared Spectroscopic Study of Cultivating Medicinal Fungi *Ganoderma*: Composition, Development, and Strain Variability of Basidiocarps

**DOI:** 10.3390/jof10010023

**Published:** 2023-12-28

**Authors:** Andriy Synytsya, Roman Bleha, Anastasia Skrynnikova, Tamilla Babayeva, Jana Čopíková, František Kvasnička, Ivan Jablonsky, Pavel Klouček

**Affiliations:** 1 Department of Carbohydrates and Cereals, University of Chemistry and Technology Prague, Technická 5, 16628 Prague, Czech Republic; as.skrynnikova@gmail.com (A.S.); mirzayet@vscht.cz (T.B.); copikovj@vscht.cz (J.Č.); 2 Department of Meat and Preservation, University of Chemistry and Technology Prague, Technická 5, 16628 Prague, Czech Republic; frantisek.kvasnicka@vscht.cz; 3 Department of Gardening, Czech University of Life Sciences Prague, Kamýcká 129, 16500 Prague, Czech Republic; i.jablonsky@seznam.cz; 4 Department of Food Science, Czech University of Life Sciences Prague, Kamýcká 129, 16500 Prague, Czech Republic; kloucek@af.czu.cz

**Keywords:** medicinal fungus *Ganoderma*, basidiocarp, ATR-FTIR spectroscopy, biochemical composition, triterpenoids

## Abstract

Attenuated total reflection Fourier-transform infrared (ATR-FTIR) spectroscopy was proposed for rapid, versatile, and non-invasive screening of *Ganoderma* basidiocarps to assess their potential for specific applications. Fifteen species and strains of this fungus were selected for analysis, and fine sections at different parts of young and mature basidiocarps were obtained. The spectra of fungal samples showed significant differences interpreted in terms of biochemical composition using characteristic bands of proteins, polysaccharides, lipids, and triterpenoids. Obviously, for the transverse sections in trama, especially in the basal part, the most intense bands at 950–1200 cm^−1^ corresponded to polysaccharide vibrations, while for the superficial sections, the bands of carbonyl and aliphatic groups of triterpenoids at 1310–1470, 1550–1740, and 2850–2980 cm^−1^ predominated. The pilei, especially hymenium tubes, apparently contained more proteins than the bases and stipes, as evidenced by the intense bands of amide vibrations at 1648 and 1545–1550 cm^−1^. The specificity of the *Ganoderma* basidiocarp is a densely pigmented surface layer rich in triterpenoids, as proved by ATR-FTIR spectroscopy. The spectral differences corresponding to the specificity of the triterpenoid composition may indicate the prospects of individual strains and species of this genus for cultivation and further use in food, cosmetics, or medicine.

## 1. Introduction

Wood-rotting polypores *Ganoderma* (family Ganodermataceae, order Polyporales) are commonly used in traditional oriental medicine. Many species of this genus are widely cultivated in the Far East region. Fungi of this genus contain proteins, polysaccharides, triterpenoids, polyphenols, and other substances with various biological effects [1,2,3], so they can be used in functional foods [4], pharmacy [5], or cosmetics [6]. The genus *Ganoderma* demonstrates a wide variety of species and strains, differing in genetics, morphology, and biochemical composition [7,8,9].

The presence and distribution of target bioactive components in the fruiting bodies (basidiocarps) and other parts of this fungus may depend on the genetic background, cultivation conditions, and developmental stage. There are many studies of cell wall macromolecules and secondary metabolites in *Ganoderma* basidiocarps based on various analytical methods, including photometric assays [10], different techniques of mass spectrometry (MS) [11,12,13,14], one- and two-dimensional (1D, 2D) nuclear magnetic resonance (NMR) spectroscopy [15,16,17,18,19], gas chromatography with MS detection (GC-MS) [20], high-performance liquid chromatography (HPLC) [10,21], and HPLC with MS [21,22,23,24] or NMR [25] detection. These methods exhibit high accuracy, selectivity, and sensitivity to the structure of the analytes. However, they have limitations related to the number of simultaneously analyzed compounds, requirements for solubility or volatility of the analytes, the invasive nature of sample preparation, or the high cost of equipment, which often makes them unsuitable for routine analysis.

There is a need for a rapid, universal, and non-invasive method to screen the biochemical composition of *Ganoderma* fungi to assess their potential for specific applications. Fourier transform infrared spectroscopy (FTIR) would be suitable for this purpose, allowing for the identification of fungal biopolymers and metabolites based on characteristic absorption bands [26,27,28]. It is a low-cost, non- or less invasive multi-analytical method, capable of in situ analysis, and highly sensitive to the sample structure. Attenuated total reflection (ATR) is a sampling technique that enables the measurement of sample surfaces, making it suitable for analyzing mushroom basidiocarp slices. FTIR microspectroscopy has been used to identify and study the intake and broken spores of *G. lucidum* [29,30,31]. FTIR and 2D infrared (2D-IR) spectroscopy have been applied to compare the composition and storage stability of basidiocarps and crude water extracts from *G. lucidum* [32,33,34], and commercial products, which supposedly come from *G. lingzhi*, were verified by the same method [35]. Screening of wild-grown and cultivated fruiting bodies and spores of *G. lucidum* based on their biochemical composition has been made by a combination of ATR-FTIR spectroscopy and multivariate discrimination analysis [36,37,38].

This work focuses on ATR-FTIR spectroscopic evaluation of the biochemical composition of young and mature basidiocarps of *Ganoderma* depending on the morphological part, location, growth stage, and species/strain. The spectra of reference organic compounds were also measured and used for comparison and band assignment.

## 2. Materials and Methods

### 2.1. Samples and Cultivation

The fifteen species and varieties of the genus *Ganoderma* were chosen from the collection of the Research Institute of Crop Production in Prague: *G. applanatum*, *G. applanatum* 2023, *G. lucidum* KZ 76, *G. lingzhi*, *G. oregonense*, *G. pffeiferi*, *G. resinaceum*, *G.* sp. 2a, *G*. sp. 2b, *G*. sp. 338, *G*. sp. 338a, *G*. sp. 338b, *G*. sp. 780, *G*. sp. KZ 74, and *G. tsugae*. These mushrooms were cultivated and harvested by the Department of Horticulture, Czech University of Life Sciences Prague (Prague, Czech Republic). Hardwood sawdust, enriched with wheat bran by 20% and supplemented with water up to 65–68%, was packaged in polypropylene bags of 2500 g each. The substrates were subjected to pasteurization at 90 °C for 24 h. Substrates were inoculated with 5% grain spawn from selected *Ganoderma* samples. The growth of the mycelium through the substrate took 21–27 days. Blocks of the overgrown substrate of various *Ganoderma* species were placed at a temperature of 25–30 °C and a relative humidity of 80–85%. Fruiting occurred under these conditions, and basidiocarps at different stages of development were collected and dried in an oven at 40 °C. The harvesting time was August 2020. Specification of the basidiocarp samples is summarized in Table 1.

Reference compounds, including protein (wheat gluten), polysaccharides (crab shell chitin, starch, and yeast *β*-d-glucan), and triterpenes (ganoderic acids A, B, and D), were purchased from Sigma Aldrich (St. Louis, MO, USA), while linseed oil originated from a local chain store. Reference (1 → 3)-*α*-d-glucan was isolated from the dried basidiocarp of cultivated mushrooms Pleurotus ostreatus obtained from mushroom grower Ing. Rudolf Ryzner (Kojátky, Czech Republic). This polysaccharide was isolated and purified according to Baeva et al. 2020 [39].

### 2.2. Analysis of the Basidiocarp Composiiton

The lower (bases), middle (stipe), and upper (pileus) parts of dried young *G*. sp. 2a (**9**) basidiocarps were separated, homogenized sequentially using an IKA A11 Basic analytical mill (IKA Werke GmbH & Co. KG, Staufen im Breisgau, Germany) and a MM 301 oscillating mill (RETSCH GmbH, Haan, Germany), and used for the analysis of the elemental and chemical composition.

#### 2.2.1. Organic Elements and Moisture

Carbon, hydrogen, nitrogen, and sulfur contents in basidiocarps were determined by using Elementar vario EL Cube equipment (Elementar Analysensysteme, Langenselbold, Germany). The Karl Fischer titration method was carried out on a Volumetric Karl Fischer titrator AF8 (Thermo Orion Inc., Waltham, MA, USA) for the determination of moisture in the homogenized samples tempered at 25 °C [40].

#### 2.2.2. Polysaccharides

The total glucans, *α*-d-glucans, and *β*-d-glucans were determined using the analytical set “MUSHROOM and YEAST β-GLUCAN” K-YBGL (Megazyme International, Wicklow, Ireland). The assay compares glucose content through the total acidic hydrolysis of glucans and the specific enzymatic hydrolysis of *α*-d-glucans [28,41,42,43]:

- Total acidic hydrolysis: samples dissolved in ice-cold concentrated hydrochloric acid underwent hydrolysis with the same acid diluted to 1.3 mol L^−1^ at 100 °C for 2 h; then a mixture of exo-(1 → 3)-*β*-glucanase and *β*-glucosidase was applied to hydrolyze *β*-glucan residues.
- Enzymatic hydrolysis of *α*-d-glucans: samples dissolved in 1.7 mol L^−1^ sodium hydroxide were hydrolyzed by amyloglucosidase in 1.2 mol L^−1^ sodium acetate buffer (pH 3.8).

The absorbance of the resulting color complex formed during the oxidation of released glucose was measured at 510 nm using an Epoch TM 2 microplate reader (BioTek Instruments, Winooski, VT, USA). The content of *β*-d-glucans was calculated as the difference between the total and *α*-d-glucans. We made all these measurements in triplicate.

The amount of chitin in the basidiocarps was determined as glucosamine via coupled capillary isotachophoresis and capillary zone electrophoresis (CITP-CZE) after acidic hydrolysis [44]. A sample (0.5 g) was hydrolyzed with 6 mol L^−1^ sulfuric acid (5 mL) in an air thermostat heated to 110 °C for 6 h. The hydrolysate was diluted with deionized water up to 10^3^ times, and glucosamine was determined as a cation using the following electrolytes:

- Leading electrolyte (pre-separation capillary, ITP step): 10 mM sodium acetate, 10 mM acetic acid, and 0.05% hydroxyethyl cellulose (pH 4.7);
- Background electrolyte (analytical capillary, ZE step): 20 mM β-alanine, 40 mM acetic acid, and 0.1% hydroxyethyl cellulose (pH 3.9);
- Terminating electrolyte: 10 mM ε-amino-*n*-caproic-acid and 5 mM acetic acid (pH 4.8).

The driving currents applied in the preseparation and analytical capillary were 250 and 75 μA, respectively. Sample hydrolysate and standard solutions were injected by a sample valve with a fixed internal sample loop (30 μL). Electrophoretic analysis used a conductometer as a detector and took 20 min. The amount of chitin (% dry matter) was represented as anhydrous *N*-acetylglucosamine (aGlcN). We made all these measurements in triplicate.

#### 2.2.3. Proteins

The protein content (% dry matter) in basidiocarps was calculated from the total (elemental) nitrogen and chitinous nitrogen [45,46]:

Proteins (% dry matter) = *N* (proteins) × 6.58 × 100/(100 − moisture), (1)

where

*N* (proteins) = *N* (total) − *N* (chitin), (2)

*N* (chitin) = aGlcN × 14/202. (3)

### 2.3. Preparation of Basidiocarp Slices

**Figure 1:**
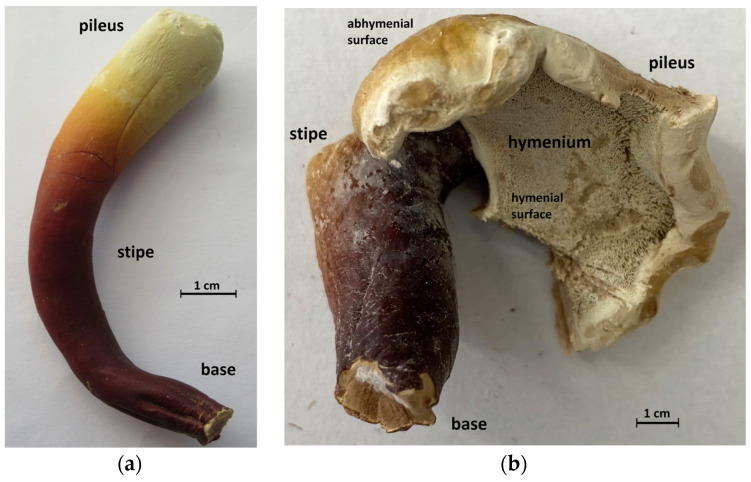
Images of *Ganoderma* basidiocarps: (**a**) dried young basidiocarp of *G.* sp. 2a; (**b**) dried mature basidiocarp of *G. applanatum* 2023 (**b**).

Thin slices of dried *Ganoderma* basidiocarps were cut with a sharp scalpel and assigned according to species and strain, age (young or mature), color (from white to dark brown), morphoanatomical part (base, stipe, or pileus), and location (trama or surfaces), as summarized in Table 1. Figure 1 demonstrates the structure of young and mature *Ganoderma* basidiocarps. In polypores, the downside of pileus carries a hymenium layer consisting of a spongy mass of downward-pointing tubes with pores. For young basidiocarps, pileus is not well differentiated (Figure 1a), while for mature pilei, upper (abhymenial) and lower (hymenial) surfaces are well developed (Figure 1b), so slices were obtained separately from these parts.

**Figure 2:**
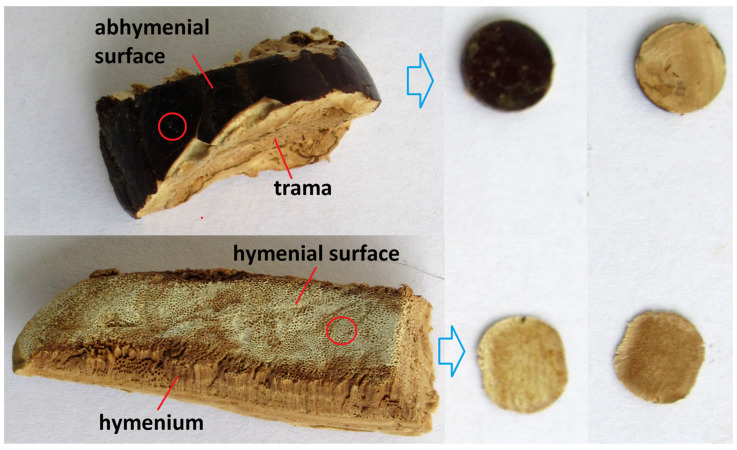
Pieces of dried pileus *G*. sp. 338a and pressed discs (50 mm diameter) prepared from the upper and lower sides of the pileus and used for ATR-FTIR recording: top (**left**) and bottom (**right**) views.

All basidiocarps were pre-dried because wet ones were difficult to measure via ATR-FTIR due to the squeezing out of water. The consistency of the dry basidiocarps was soft enough for further manipulation. We tried to take both surface and internal sections for subsequent measurements. Our experience has shown that for better contact with the surface of the ATR crystal, it is necessary that the cut be sufficiently dense, not springy, and that its surface be even and smooth. It was sometimes hard to make good contact with the ATR crystal, especially when analyzing mature basidiocarps. For this purpose, the superficial sections from both sides of the pileus were pressed with the hand press (Pike Technologies, Madison, WI, USA) into discs with faces corresponding to the abhymenial surface, trama, hymenial surface, and hymenium inside (Figure 2). We noticed that a section taken from the surface of the basidiocarp has the spectra on both sides being very different because they corresponded to the composition of the surface and the interior. Therefore, one such disc met the requirements of two samples at once—from the surface and the inside. This approach simplifies the work with the raw material, and we offer it for routine measurements. The spectra measured from such discs and the corresponding sections were practically the same, although the intensity of the signal depended on the degree of contact of the sample with the surface of the crystal, which in the case of a disc often exceeded that of ordinary sections.

### 2.4. Recording of ATR-FTIR Spectra

ATR-FTIR spectra of the basidiocarp slices and reference compounds were recorded on a Nicolet 6700 FTIR spectrometer (Thermo Fisher Scientific, Waltham, MA, USA) using the HATR accessory (ZnSe crystal) with a resolution of 2 cm^−1^ in the ranges of 4000–600 cm^−1^; 64 scans were collected for each spectrum. The spectra were successively smoothed (9–11 ppt), ATR corrected, and baseline-corrected using Omnic 8.0 software (Thermo Fisher Scientific, Waltham, MA, USA). Then, the spectra were exported in ASCII format to Origin 6.0 (OriginLab, Northampton, MA, USA) software to prepare output graphs. The second derivative analysis of the corrected ATR-FTIR spectra was made using Omnic 8.0 software using the first difference option. The obtained second derivatives of the initial spectra were applied to detect a position of overlapping bands (shoulders).

## 3. Results and Discussion

ATR-FTIR spectra of the slices obtained from dried *Ganoderma* basidiocarps showed significant differences depending on the origin, age, and morphoanatomical part from which they were cut. The spectra were analyzed for the presence of bands characteristic of the main components of the basidiocarp, namely proteins, polysaccharides, lipids, and triterpenoids. Figures S1 and S2 show the ATR-FTIR spectra of the reference compounds for comparison, which represent fats (linseed oil), proteins (gluten), and polysaccharides (chitin, starch, and fungal d-glucans). The spectral contribution of other compounds of *Ganoderma* basidiocarps, such as polyphenols and pigments (melanin), was much less pronounced or negligible, so we focused on the mentioned main compounds.

### 3.1. ATR-FTIR Spectra of Young Basidiocarps

In young basidiocarps of *Ganoderma* samples **6**, **9**, **10**, **13**, and **14**, the upper and younger part (pileus) was poorly differentiated but often less pigmented. Samples were taken from the lower, middle, and upper parts, assigned as base, stipe, and pileus, respectively, from the inside (trama) and the surface layers.

#### 3.1.1. Composition of Trama

**Figure 3:**
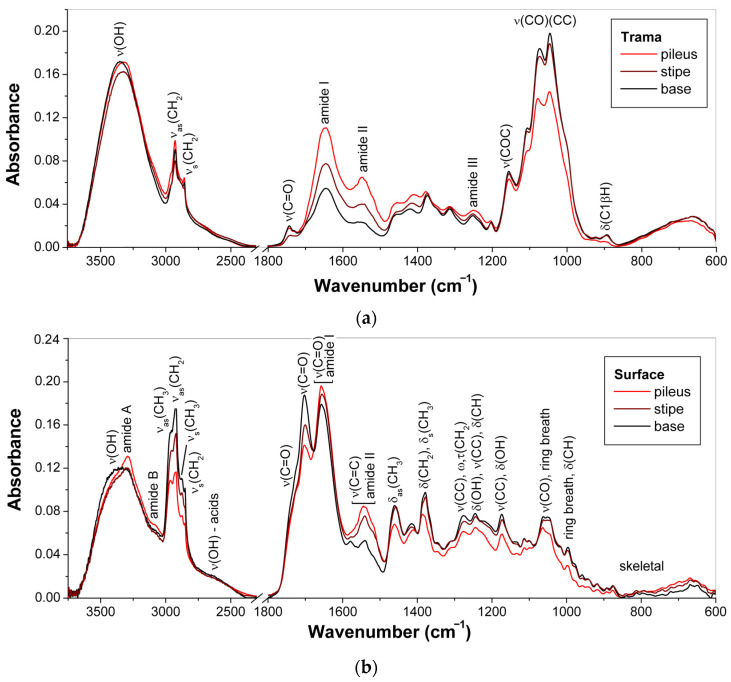
ATR-FTIR spectra of dried young basidiocarps of *G*. sp. 2a: (**a**) trama; (**b**) surface layers.

Figure 3 represents the ATR-FTIR spectra of trama, the inner fleshy mass, and surface layers of young basidiocarps of *G*. sp. 2a (**9**), separately base, stipe, and pileus. The obtained spectra of trama (Figure 3a) closely resemble the spectrum of *G. lucidum* raw material in dry powder form, as previously published [33], because trama makes up the bulk mass, which means that its composition approximately corresponds to the composition of the entire basidiocarp. By comparing the intensity of the characteristic bands in the ATP-FTIR spectra of the trama obtained from the base, stipe, and pileus, one can indicate the ratio of biopolymers in these morphoanatomical parts.

For the ATR-FTIR spectra of trama, the highly overlapped intense bands of C–O–C, C–O, and C–C stretching vibrations at 1154, 1105, 1072, 1044, and 995 cm^−1^ (shoulder) and the weak band of C1β–H bending at 891 cm^−1^ confirmed the predominance of polysaccharides, mainly *β*-d-glucans [15,28,47,48,49], but also chitin and *α*-d-glucans. The spectrum of yeast *β*-d-glucan (Figure S1) showed the corresponding bands at very similar wavenumbers. The O–H stretching vibrations of polysaccharides contribute to the broad band at 3317–3345 cm^−1^ [50], together with the O–H stretching vibrations of water [51], polyphenols [52], and some triterpenoids [53,54], and the O–H and N–H stretching vibrations of proteins [55]. These bands subsequently decreased in the order base, stipe, and pileus. On the other hand, the less pronounced bands of amide I, amide II, and amide III vibrations observed at 1643, 1547, and 1228 cm^−1^ (shoulder), respectively, indicated the presence of proteins [33,56]. These bands grew in the opposite order than the polysaccharide bands mentioned above. In addition, weak bands at 2923, 2853, and 1741 cm^−1^ arose from CH_2_ and C=O stretching vibrations of lipids [57]. These lipid bands were the lowest for the stipe.

The chemical composition of the parts of dried young *G*. sp. 2a (**9**) basidiocarps (bases, stipes, and pilei) was determined by the convenient methods (see Section 2.2) to confirm the spectral patterns of proteins and polysaccharides. Table 2 summarizes the results obtained. Pilei showed the highest nitrogen (6.37% *w*/*w*) and sulfur (0.37% *w*/*w*) levels in basidiocarps explained by increased protein content. However, the difference between bases and stipes is not so evident, and bases showed an even higher amount of these elements than stipes. Chitin, a linear (1 → 4)-2-acetamido-2-deoxy-*β*-d-glucan, is another principal source of fungal nitrogen [46], and its amount was maximal for bases (15.5% in dry matter) that corresponded to 0.93% *w*/*w* of chitinous nitrogen. The remainder of nitrogen (1.33% *w*/*w*) originated mainly from proteins, which comprised ~10% in dry matter. It was the lowest value among the basidiocarp parts, and the proteins grow in the order bases, stipes, and pilei up to ~44%. By contrast, total d-glucans and *β*-d-glucans were maximal for bases (~55% and ~45% in dry matter) and gradually decreased in the same order. These trends in the amounts of proteins and glucans confirmed the results obtained via ATR-FTIR based on the relative intensities of characteristic bands.

The increased content of proteins in the pileus is because this is the youngest part of the basidiocarp, where intensive biosyntheses occur associated with the growth and development of hymenium and spores for reproduction. On the contrary, the base and the stipe primarily have the mechanical function of holding the pileus, which requires polysaccharides as the main structural components of cell walls. Chitins and *β*-d-glucans dominate the base, anchoring the entire basidiocarp to the substrate.

The study of *G. lingzhi* at different growth stages demonstrated that the highest amount of polysaccharides extractable with water was determined in young basidiocarps at the early stages of development [58]. A similar study on *G. lucidum* basidiocarps demonstrated that the total content of soluble polysaccharides, including (1 → 4)-*α*-d-glucan and (1 → 3)-*β*-d-glucan decreased after the primordium developed into a fruiting body [59]. However, only a minor water-soluble fraction of polysaccharides is extractable with hot water, while the rest, primarily (1 → 3)-*α*-d-glucans, (1 → 3)(1 → 6)-*β*-d-glucan and chitin, remain in the solid state [15]. Consequently, the decrease in the yield of water-soluble polysaccharides at the last stages of basidiocarp development is associated with the chitin–glucan complex strengthening, not with a decrease in total cell wall polysaccharides.

#### 3.1.2. Composition of Surface Layer

The name of the genus *Ganoderma* can be translated as “glossy leather”, reflecting a principal feature of the basidiocarp morphology. The presence of a dense and shiny cuticle has a protective significance, which, undoubtedly, should be reflected in its biochemical composition. Unfortunately, we could not find any studies which specifically address this topic. This surface layer arises during the development of the basidiocarp and accumulates bioactive compounds, protecting the organism against external influences, especially attacks from pathogenic microorganisms. Triterpenoids and pigments are two classes of *Ganoderma* metabolites known by many biological activities [60,61] and thus can perform a protective function at the basidiocarp surface. Although some triterpenoids can inhibit tyrosinase (EC 1.14.18.1), a key enzyme of melanin biosynthesis [62,63], there may be some antagonism between these compounds.

Vibrational spectra of ganoderic acids, the main *Ganoderma* triterpenoids, were recently described and interpreted using DFT calculations [64,65]. The region of 1500–1800 cm^−1^, assigned to the stretching vibrations of the C=O and C=C bonds, has been used for the identification of various ganoderic acids differing in the number and position of the carbonyl, carboxylic, or ester groups attached to the rings or open chain [53,54,65,66,67,68,69]. On the other hand, comparing the band intensity in the regions of 3000–2800 cm^−1^ (C–H stretching) and 1500–1800 cm^−1^ (C=O stretching) is informative to the ratio between acidic and neutral triterpenoids. Indeed, only the spectra observed for acidic forms show an intense C=O stretching band of carboxylic groups around 1700 cm^−1^, while the spectra observed for all triterpenoids have several strong IR bands of C–H stretching vibrations due to the contribution of the hydrocarbon skeleton having CH_2_ and C–H groups in the rings and open chain and CH_3_ groups as substituents. Comparing the intensities of individual C–H stretching bands helps distinguish the relationship between these groups in differently substituted triterpenoids.

In contrast to those mentioned above for the trama (Section 3.1), the ATR-FTIR spectra of the surface slices of *Ganoderma* basidiocarps (Figure 3b) have several intense bands observed at ~2850–2960, 1540–1800, and 1380–1460 cm^–1^ and assigned to C–H stretching, C=O stretching, and C–H bending vibrations of triterpenoids, respectively [53,54,65,66,67,68,69]; the bands observed below 1300 cm^–1^ corresponded to the C–C stretch, open chain, and ring vibrations of these compounds [65,68,69]. The mentioned bands were the most pronounced for the surface layer located in the base. For the surface slices taken from pilei and stipes, the characteristic bands of amide vibrations at 3295 cm^−1^ (amide A), ~3080 cm^−1^ (amide B), ~1657 cm^−1^ (amide I, overlapped by the intense band of C=O stretch in triterpenes), and 1541–1546 cm^−1^ (amide II, overlapped by the band of C=C stretch in triterpenes) were more pronounced due to the higher contribution of proteins [33,56]. As seen in Figure 1a, for young basidiocarps of *G*. sp. 2a (**9**), these differences correlated with color intensity from white (pileus) and orange (stipe) to dark brown (base), probably due to the presence of 3,4-dihydroxyphenylalanine melanin from *G. lucidum* basidiocarps [60,70]. However, no vibration bands indicating the presence of melanin were detected.

ATR-FTIR spectra thus confirmed that the surface layer of the young *Ganoderma* basidiocarp has a composition different from that of its trama. Triterpenoids, proteins, and pigments are concentrated in the peripheral part, affecting spectra and color. As in trama, the contribution of proteins in the surfaces gradually decreased from the pileus to the stipe and base, but the contribution of triterpenoids and associated pigments increased in this row.

Nakagawa et al. 2018 and Zhou et al. 2018 [58,59] studied the composition of *Ganoderma* basidiocarps at different growth stages. They observed the highest total triterpenoids extracted with ethanol in young basidiocarps at the stages of stipe elongation or early development of pileus. Moreover, the composition of triterpenoids also showed dependence on the growth stage, so their profiles for young and mature basidiocarps were quite different. Perhaps at these stages, the basidiocarp is most sensitive to external influences, and the increased production of specific triterpenoids is protective.

### 3.2. ATR-FTIR Spectra of Mature Basidiocarps

Mature basidiocarps of *Ganoderma* samples **1**–**5**, **7**, **8**, **11**, **12**, and **15** had pilei with a well-developed hymenium. Samples were taken from the upper and lower parts and the inside of the pileus. The manufacture of pressed discs made it possible to distinguish between the outer and inner faces of the same sample. Furthermore, we compared regions with different levels of pigmentation.

**Figure 4:**
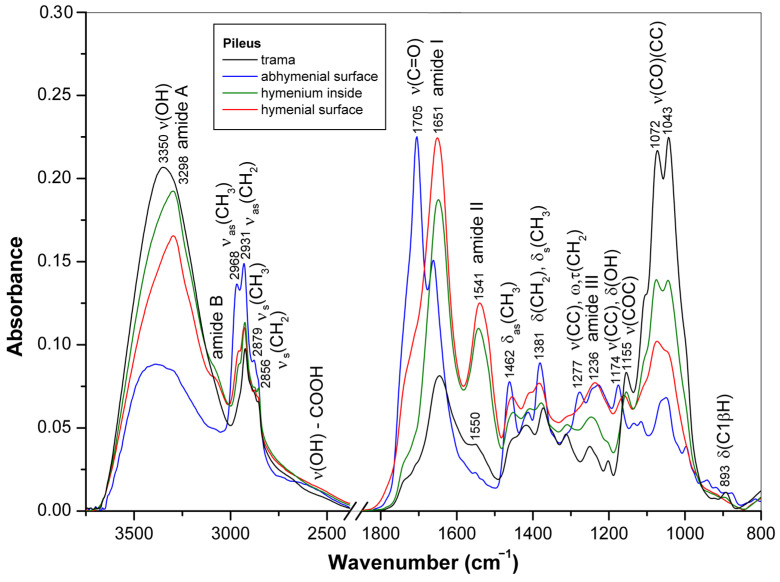
ATR-FTIR spectra of various parts of dried mature basidiocarp (pileus) of *G*. sp. 338a.

The ATR-FTIR spectra of the pressed discs obtained from dried mature basidiocarp of *G*. sp. 338a (**12**) represented various parts of the pileus, i.e., trama, hymenium, abhymenial and hymenial surfaces, are shown in Figure 4. As in the case of young basidiocarp (Section 3.1), the intense characteristic bands in the spectra of these discs showed that polysaccharides predominated in the trama, but triterpenoids and proteins were the main components of the abhymenial and hymeneal surfaces, respectively. The characteristic protein and polysaccharide bands observed in the spectrum of hymenium were of comparable intensities and coincided with the bands of reference compounds (Figure S1). The bands observed in the spectrum for trama at 1417, 1373, 1313, 1250, 1201, 1155, 1072, 1043, and 893 cm^−1^ originated mainly from (1 → 3)(1 → 6)-*β*-d-glucan [15,47,48,49]. The shoulder at 1550 cm^−1^ was assigned to the amide II vibration in chitin [46]. The spectra of hymenium and its surface demonstrate protein bands at 3298, 3080, 1649–1651, 1541–1543, and 1236 cm^−1^ [33,56]. Finally, the bands observed for abhymenial surface at 2968, 2931, 2879, 1705, 1662, 1462, 1411, 1381, 1277, 1228, 1174, 1134, 1117, 1050, 997, 943, 920, 877, and 816 cm^−1^ indicated the presence of triterpenoids [53,54,65,66,67,68,69]; see Table S2 for assignments. Therefore, ATR-FTIR spectra of mature basidiocarps also confirmed the assumption that triterpenoids of fungi *Ganoderma* are located mainly in the abhymenial surface layer of basidiocarps, and the relative amount of these compounds compared to other major biochemical constituents, such as polysaccharides or proteins, depends on the stage of development and the morphoanatomical part of the basidiocarp.

The prevalence of proteins in the hymenium compared to trama is because it is in this part of the basidiocarp that spores develop and mature due to increasing biosynthesis involving numerous enzymes. On the contrary, the upper (abhymenial) pileus layer protects the hymenium, so there are more triterpenoids with antimicrobial and other biological activities. Structural polysaccharides prevailed in the stroma, making up the cell walls and determining the structure of the entire basidiocarp. For most samples of *Ganoderma* strains and species, ATR-FTIR spectra demonstrated the distribution of the main biochemical constituents of the pileus similar to that mentioned above. However, this was not the case with *G. applanatum* (**2**) and *G. lucidum* KZ 76 (**3**) having pilei with weakly pigmented abhymenial and hymenial surfaces. In some forms of *Ganoderma*, the biosynthesis and distribution of pigments and triterpenoids associated with them may be disturbed due to mutations or environmental and growing conditions.

**Figure 5:**
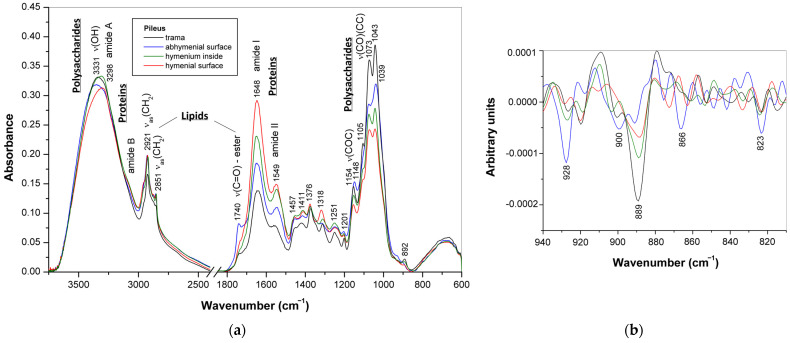
(**a**) ATR-FTIR spectra of various parts of dried mature basidiocarp (pileus) of *G. applanatum*; (**b**) second derivatives of these spectra in the region of carbohydrate vibrations sensitive to the anomeric structure.

The FTIR spectra observed for the pilei of strains **2** and **3** did not have characteristic bands of triterpenoids and mainly exhibited protein and polysaccharide bands in varying proportions for different parts of the pileus. As shown in Figure 5a, the intensity of protein bands, observed for strain **2**, at 1648 and 1545–1550 cm^−1^ [33,56] increased, and the contribution of polysaccharide bands at 1148–1154, 1105, 1073–1075, 1039–1043, and 892 cm^−1^ [15,47,48,49] decreased in the order of trama, abhymenial surface, hymenium, and hymenial surface. The difference in the polysaccharide composition between the abhymenial surface and other parts of the pileus is also indicated by the shift in the bands observed for the trama at 1154 and 1043 cm^−1^ to lower frequencies, i.e., 1148 and 1039 cm^−1^, respectively. The wavenumber of the broad O–H stretching band shifted from 3331 cm^−1^ (trama) to 3298 cm^−1^ (hymenial surface) due to the contribution of the amide A vibration in proteins [56]. The bands at 2920–2927, 2851–2857, and 1740 cm^−1^ indicate the presence of lipids, which were more pronounced for the abhymenial surface [57]. For comparison, the corresponding bands for flaxseed oil were at 2925, 2854, and 1745 cm^−1^ (Figure S1).

The second derivatives of the spectra at 810–940 cm^−1^ indicate weak polysaccharide bands sensitive to the anomeric configuration of the carbohydrate units (Figure 5b). The band at ~890 cm^−1^ was most intense for trama and gradually decreased in the order hymenium, hymenial surface, and abhymenial surface. Its position is specific for 1,3-linked *β*-d-glucosyl residues [47,71] and thus indicates the predominance of (1 → 3)-*β*-d-glucan in trama. On the contrary, the bands of *α*-d-glucans at 823, 866, and 928 cm^−1^ [46,72] and the band of chitin at ~900 cm^−1^ [46] were more pronounced for the abhymenial surface. The similar bands of reference polysaccharides were at 821 cm^−1^ (fungal *α*-d-glucan), 861 cm^−1^ (starch), 898 cm^−1^ (chitin), and 930–931 cm^−1^ (*α*-d-glucans) (Figure S1).

Cell wall polysaccharides maintain the structural integrity of the fungal organism by providing the necessary balance between hardness and elasticity, so the content and distribution of chitin and glucans in the *Ganoderma* basidiocarp will depend on the physiological role and metabolism of its morphological parts.

### 3.3. Triterpenoids in Surface Layer of Ganoderma Basidiocarps

Basidiocarps, mycelia, and spores of *Ganoderma* are known as the source of biologically active lanostane-type triterpenoids [17,18,19,60,73,74,75]. These compounds are structurally diverse and include acids and alcohols. Acids have a carboxylic group in the side chain, while alcohols do not. Among the former, the most abundant ganoderic and lucidenic acids [76], as well as many others, i.e., ganohainanic, ganolucidic, ganosporeric, and hainanic acids [75,77], differ in the detailed skeleton structure and the location of substituents.

Triterpenoids play a crucial role in protecting the fungus from external influences, and their overproduction is a strategy of the *Ganoderma* in the fight against infections and parasites [78], so it is not surprising that these substances are concentrated in the superficial layers of the basidiocarp, i.e., in the first line of defense and contact with the environment. The current section focuses on the FTIR spectroscopic profiles of triterpenoids in different morphological parts of *Ganoderma* basidiocarps, particularly in the surface layers (where these compounds might predominate) for various *Ganoderma* species and strains. The IR band assignments for the surface slices rich in triterpenoids are summarized in Table S2 compared with the corresponding bands of reference ganoderic acids A, B, and D, the main *Ganoderma* triterpenoids.

#### 3.3.1. Triterpenoid Distribution in *Ganoderma* Basidiocarps

FTIR spectroscopy makes it possible to differentiate such structurally similar plant triterpenoids as betulin, betulinic acid, and lupeol [79,80]. This method distinguishes individual triterpenoids and evaluates their quantitative ratios for raw bark and extracts. Similarly, for *Ganoderma* fungi, FTIR spectroscopy can be used to analyze the distribution of triterpenoids in the surface layer of basidiocarps, where these compounds often dominate over other biochemical components.

For most samples of *Ganoderma* basidiocarps, the bands attributed to triterpenoids prevailed in the ATR-FTIR spectra measured for the surface layers of various localizations, i.e., in the bases, stipes, or pilei, including the abhymenial and hymenial sides of mature pilei. However, the spectra revealed significant differences in the composition of these surface layers, which were associated not so much with the presence of proteins or polysaccharides but, above all, with the specific composition of the triterpenoids. The distribution of triterpenoids in different sites of the same basidiocarp may be associated with its growth and developmental features and with some special functions of its morphological parts.

**Figure 6:**
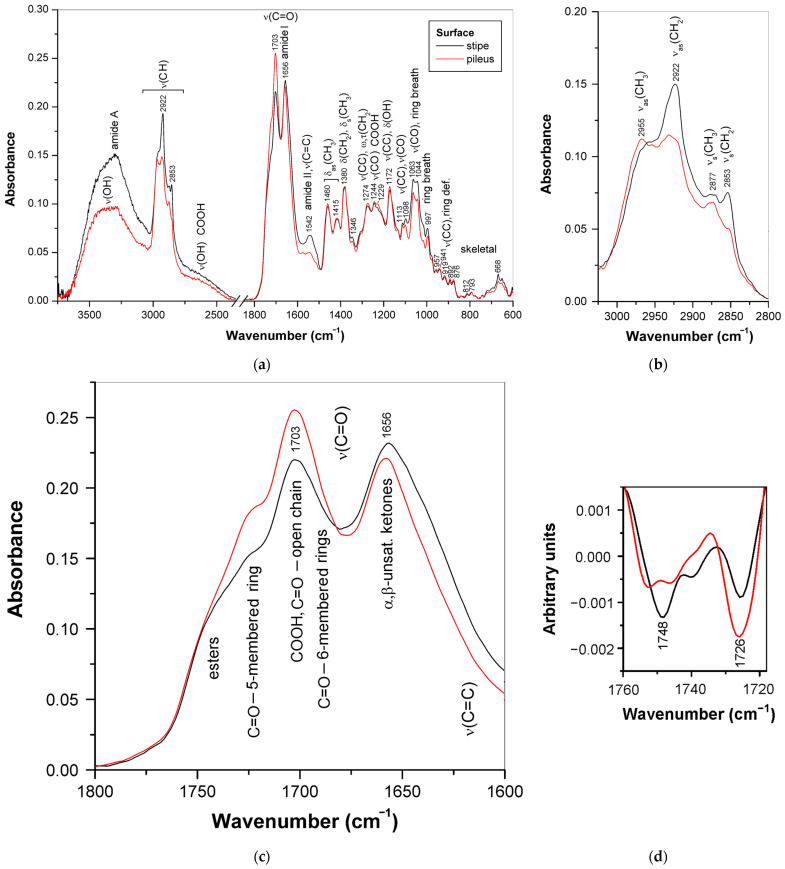
ATR-FTIR spectra of the surfaces of stipe and abhymenial side of pileus of dried mature basidiocarp of *G. applanatum* 2023: (**a**) the whole spectra; (**b**) the CH stretching region; (**c**) the C=O stretching region; (**d**) the second derivatives of the spectra at the C=O stretching region.

**Figure 7:**
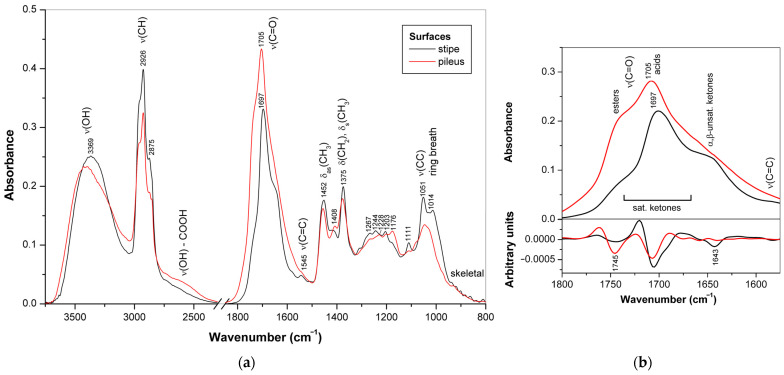
ATR-FTIR spectra of the surfaces of stipe and abhymenial side of pileus of dried mature basidiocarp of *G. resinaceum*: (**a**) the whole spectra; (**b**) the C=O stretching region including spectra (**top**) and second derivatives (**bottom**).

The ATR-FTIR spectra of the surface layers cut off from the stipe and the abhymenial side of pilei from dried basidiocarps of *G. applanatum* 2023 (**1**) and *G. resinaceum* (**7**) are shown in Figure 6a and Figure 7a, respectively. Despite some weak protein features, the spectra measured for strain **1** represent the purest triterpenoid bands among all basidiocarp samples measured in this study, and even weak skeletal vibration bands of these compounds are visible below 900 cm^−1^.

The spectra related to the stipe and pileus surfaces are very similar, and many bands starting from 1460 cm^−1^ and below practically coincide in their position and relative intensity, which confirms the structural basis of the lanostane-type triterpenoids. The regions of C–H and C=O stretching vibrations showed the highest differences. These vibrations are sensitive to the specificity of individual triterpenoids. In the C–H stretch region (Figure 6b), the bands of CH_2_ groups at 2922 and 2853 cm^−1^ prevailed for the stipe surface and were much less pronounced for the abhymenial surface of the pileus, having little more pronounced bands of CH_3_ groups at 2955 and 2877 cm^−1^ [81].

For the C=O stretching region (Figure 6c), the bands at 1703 and 1656 cm^−1^ were the strongest. The former band belongs to the carboxylic and carbonyl groups attached to the open chain, and the latter band belongs mainly to the α,β-unsaturated carbonyl groups [53,54,65] with some contribution by protein vibration amide I. For the abhymenial surface layer of the pileus, the band at 1703 cm^−1^ was the most intense in the spectrum, whereas for the surface of the stipe, this band was less pronounced and comparable in its intensity with the neighbor band at 1656 cm^−1^. In addition, it is evident from the second derivatives of the spectra (Figure 6d) that the shoulders approximately at 1726 and 1748 cm^−1^ showed differences in their relative intensities: the former was more pronounced for the pileus, and the latter for the stipe. These shoulders were assigned respectively to 5-membered ring carbonyl [65] and ester groups [53]. The C–O stretching band observed for the abhymenial surface layer of pileus at 1229 cm^−1^ also confirms the higher contribution of carboxyl groups.

In contrast to that mentioned above for strain **1**, the profiles of the C–H stretching region in the FTIR spectra of the stipe and pileus surfaces of *G. resinaceum* (7) basidiocarp were quite similar (Figure 7a). However, the ratio between the strong bands at 2926 and 1697–1705 cm^−1^, attributed to the stretching vibrations of C–H and C=O bonds, respectively, showed significant differences. The former band was more intense for the stipe surface and the latter for the abhymenial surface of the pileus. In addition, the IR bands attributed to the hydrocarbon moieties of triterpenoids and observed at 1452, 1375, 1267, 1244, 1203, 1111, 1051, and 1014 cm^−1^ [53,54,65], as well as several weak low-frequency bands of skeletal vibrations observed below 940 cm^−1^, were more pronounced in the case of the stipe surface. By contrast, the bands associated with COO-, C–O, and O–H vibrations were observed at 1408, 1228, and 1176 cm^−1^ [65] for the abhymenial surface of the pileus. Therefore, for strain **7**, neutral triterpenoids prevailed in the stipe surface layer, while acidic terpenoids were more abundant in the abhymenial surface layer of the pileus. The most intense band of the C=O stretching vibration was at 1697 cm^−1^ for the stipe surface and at 1705 cm^−1^ for the abhymenial surface of the pileus. The shift of this band towards lower frequencies is most likely associated with a decrease in the proportion of carboxyl groups and, consequently, with the predominance of neutral terpenoids. All terpenoids, acidic and neutral, have carbonyl groups that absorb over a wider wavenumber region depending on the location in the molecule. The decrease in the intensity of the broad shoulder at about 2600 cm^−1^, which corresponds to the O–H stretching in carboxyl groups bonded by hydrogen bonds [82], confirms this assumption. In addition, these spectra differ in the intensity of the shoulders observed at about 1745 and 1643 cm^−1^, which were attributed to the C=O stretching vibrations of esters and α,β-unsaturated ketones, respectively [53,65]. It means that for *G. resinaceum*, the stipe surface terpenoids contain more α,β-unsaturated carbonyl groups, while the abhymenial surface terpenoids contain more ester groups.

#### 3.3.2. Differences in Triterpenoid Composition for the *Ganoderma* Strains and Species

**Figure 8:**
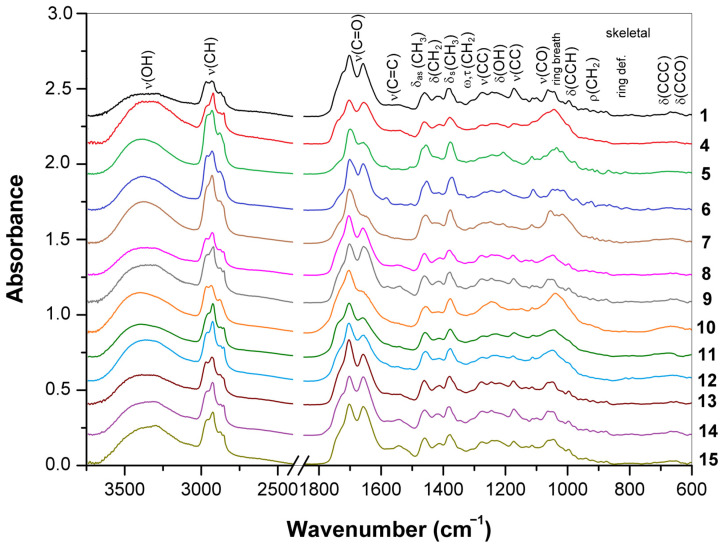
ATR-FTIR spectra of the triterpenoid-rich surface layers of basidiocarps originated from various species and strains of the genus *Ganoderma*.

FTIR spectra obtained for the triterpenoid-rich surface layers of basidiocarps represent species and strains **1**, **4**–**15** of the genus *Ganoderma* (Figure 8); the band assignment is summarized in Table S2 in comparison with the data obtained for the reference ganoderic acids A, B, and D [34,65,83,84]. For all these samples, characteristic bands of triterpenoids predominated in the spectra, which confirmed that these substances are the main constituents of the basidiocarp surface layers. In some cases, the amide II band of proteins at 1540 cm^−1^ (strains **4**, **8**, **9**, **14**, and **15**) and bands of C–O and C–C stretching vibrations of polysaccharides at 950–1200 cm^−1^ (strains **4**, **5**, **7**, **10**–**12**) overlapped triterpenoid features.

The O–H stretching region (3500–3000 cm^−1^). All spectra obtained for the triterpenoid-rich surface layers showed a broad band centered at 3300–3400 cm^−1^. This band arose from the O–H stretching vibrations of water and hydroxyl groups involved in the intermolecular hydrogen bonds. Corresponding bands of ganoderic acids were within this region. However, a sharp band at 3452–3536 cm^−1^ observed for reference ganoderic acids was absent in the spectra of the mentioned samples. This narrow signal is characteristic of an isolated hydroxyl group that does not participate in the hydrogen bonds between molecules, which may be attributive to crystalline forms of pure terpenoids. In basidiocarp samples, these molecules are closely related to other biochemical components, and hydroxyl groups may participate in the intermolecular interactions. Therefore, this narrow band found in the spectra of pure ganoderic acids is absent in the spectra of the samples. In addition, the bands or shoulders near 3295 cm^−1^ (amide A) and 3080 cm^−1^ (amide B) appeared in some cases due to the presence of proteins [56], and a broad shoulder around 2650 cm^−1^ arose from the O–H stretching vibrations of carboxylic groups [82].

The C–H stretching region (2800–3000 cm^−1^). Four bands originated mainly from the vibrations in the hydrocarbon skeleton of triterpenoids were observed at ~2950–2980 cm^−1^ (CH_3_ antisymmetric stretching), 2920–2940 cm^−1^ (CH_2_ antisymmetric stretching), 2870–2880 cm^−1^ (CH_3_ symmetric stretching), and 2850–2860 cm^−1^ (CH_2_ symmetric stretching) [64,77]. The methylene bands were usually more intense than the corresponding methyl bands, while, for samples **1**, **5**, **6**, **10**, and **13**, these bands were close in intensity. The differences in the mentioned C–H stretching bands indicate the structural features of the triterpenoids that could be characteristic of the *Ganoderma* strains and species.

The C=O and C=C stretching region (1500–1800 cm^−1^). In this region, all samples have several intense overlap bands, but their intensities vary between species and strains. There are usually two strong bands at 1699–1705 cm^−1^ and 1655–1662 cm^−1^, except the spectra for strains **7** and **10** with the single dominant band at ~1704 cm^−1^. The carboxyl and carbonyl groups in the open chain of acid triterpenoids mainly contribute to this band. For neutral triterpenoids, the band around 1710 cm^−1^ is weakened [83] or absent altogether if the molecule does not have these groups [79,80,85,86,87]. Therefore, the weakening or strengthening of this band compared to the CH_3_ and CH_2_ stretching bands mentioned above can be explained by the predominance of neutral and acidic triterpenoids, respectively. For many samples, the C–H and C=O stretching bands are comparable in intensity, but for strains **1**, **8**, **10**, and **13**, the bands of carbonyl groups were much more pronounced, which indicates the predominance of acid triterpenoids. Conversely, for strains **5** and **7**, the intensity of the C–H stretching bands significantly exceeds the intensity of the corresponding carbonyl stretching bands, so neutral triterpenoids were abandoned here. A weak band or shoulder observed for some strains at 1578–1586 cm^−1^ was assigned to the C=C stretching vibration [65].

The C–H bending region (1300–1500 cm^−1^). This region has two strong-to-medium bands at 1456–1462 and 1373–1380 cm^−1^ and several smaller bands or shoulders attributed mainly to CH_3_ bending and CH_2_ scissoring vibrations [65,81,85,88,89]. The splitting of methyl vibrations into several bands is due to the different positions of these groups in the molecule. The bands of angular methyls, especially those located at the junction of five- and six-member rings, shift to higher frequencies from the corresponding bands of substituents with cyclic or open chains [86,89]. In the spectra obtained for the basidiocarp surfaces, the position of these bands varied significantly. For example, for strain 6, three bands were found at 1455, 1431, and 1373 cm^−1^, while the spectrum of strain **13** showed corresponding bands at 1462, 1428, and 1379 cm^−1^. Such differences indicate the structural features of triterpenoids associated with the number and position of CH_3_ and CH_2_ groups in the molecule. Considering that the triterpenoids of *Ganoderma* fungi have a lanostane skeleton with a defined position of CH_3_ groups [61,77], the differences in the intensity and position of these bands will be affected by the contribution of additional methyl groups, for example, in acetyl esters, as well as by other vibrations, such as C–H or O–H bending, characteristic of this spectral region. Certainly, vibrations of proteins and polysaccharides can also affect this spectral region.

The C–C and C–O stretching region (1000–1300 cm^−1^). Several bands in this region observed at ~1270–1277, 1243–1247, and 1172–1175 cm^–1^ corresponded mainly to C–C stretching and, to a lesser extent, to O–H bending vibrations. The first band arises due to the stretching vibration of the ring C–C bond conjugated with the neighboring double bonds C=O and C=C. As a result, it shifted toward a higher wavenumber compared to other C–C stretch bands [65]. The intensity of the last band at 1172–1175 cm^–1^ varied significantly among strains and was very weak or absent in the spectra of strains 5–7 and 10. Several other bands observed for strains **1**, **8**, **9**, and **13**–**14** at 1133–1140, 1113–1116, 1062–1065, 1038–1046, and 1012–1018 cm^–1^ corresponded to those of the reference ganoderic acids observed at similar positions. These bands originated from the C–O stretching, C–CH_3_ waging, and ring vibrations [65]. The current spectral region was significantly different for the remaining strains due to the overlap by vibration bands of other chemical components, including polysaccharides.

The region of skeletal vibrations (600–1000 cm^−1^). Below 1000 cm^−1^, a medium band observed at ~997 cm^−1^ for strains 1, 8, 9, and 13–14 and the reference ganoderic acids was attributed to the ring breathing vibrations [65]. Due to its stability, this band can prove the presence of triterpenes, along with the previously described intense vibration bands of the carbonyl and methyl groups. Several weaker bands of skeletal vibrations observed at 790–960 cm^−1^ showed variable intensity and positions for both strains and reference compounds. Finally, several weak bands observed at 640–740 cm^−1^ were assigned to ring and open chain deformations [65]. These bands are difficult to use in triterpenoid analysis due to their low intensity and possible overlap with low-frequency bands of other biochemical components of *Ganoderma* basidiocarp.

## 4. Conclusions

ATR-FTIR spectroscopy provides insight into the content of major biochemical compounds found in *Ganoderma* basidiocarps of specific species or strains at a particular developmental stage and in a distinct morphoanatomical part. The distribution of biological macromolecules changes during the development of the basidiocarp. The pilei of young basidiocarp contained more proteins necessary for growth than the base and stipe, as evidenced by the intense bands of amide vibrations at ~1648 and 1545–1550 cm^−1^. Furthermore, during the development, there is a gradual increase in the content of cell wall polysaccharides, including (1 → 3)(1 → 6)-*β*-d-glucan, (1 → 3)-*α*-d-glucan and chitin, in the lower parts of the basidiocarp detected by the envelope of intense highly overlapping C–O–C, C–O, and C–C stretching bands at 950–1200 cm^−1^.

The specificity of *Ganoderma* basidiocarps is the gradual formation of a solid surface layer rich in triterpenoids especially pronounced at the abhymenial surface of mature pilei, while the hymenial surface and hymenium tubes mainly contain proteins. Triterpenoids of basidiocarp surface layers are especially interesting due to their potent biological activities. Marked spectral differences in triterpenoids found in the individual species and strains of the genus *Ganoderma* might indicate prospects for cultivation and use in nutrition, pharmacy, or cosmetics.

## Figures and Tables

**Table 1:** Specification of the dried basidiocarp samples used in this study *.

Sample			Basidiocarps—Slices										
No	Species	Strains	Young	Mature	Base		Stipes		Pilei				
									Surface				
					Surface	Trama	Surface	Trama	Non-differentiated	Abhymenial	Hymenial	Trama	Hymenium
**1**	*G. applanatum*	2023		+			+	+		+	+	+	
**2**	*G. applanatum*			+				+		+	+	+	+
**3**	*G. lucidum*	KZ 76		+						+	+	+	+
**4**	*G. lingzhi*			+				+		+	+	+	
**5**	*G. oregonense*			+	+	+				+	+	+	
**6**	*G. pffeiferi*		+		+	+			+			+	
**7**	*G. resinaceum*			+		+	+	+		+	+	+	+
**8**	*G. tsugae*			+	+	+	+	+					
**9**	*G.* sp.	2a	+		+	+	+	+	+			+	
**10**	*G.* sp.	2b	+				+	+					
**11**	*G*. sp.	338		+						+		+	
**12**	*G.* sp.	338a		+						+		+	
**13**	*G*. sp.	338b	+				+	+	+			+	
**14**	*G*. sp.	780	+		+	+	+	+	+			+	
**15**	*G*. sp.	KZ 74		+			+	+		+		+	

* The sign “+” indicates available samples.

**Table 2:** Elemental and chemical composition of bases, stipes, and pilei of dried young basidiocarps of strain **9**.

Analytical Method	Contents (% *w*/*w*)	Parts of Basidiocarps		
		Bases	Stipes	Pilei
Organic elemental analysis (OEA)	N	2.26	1.81	6.37
	C	47.46	51.66	46.78
	H	6.74	7.19	6.99
	S	0.17	0.15	0.37
Karl Fischer titration	Moisture	11.83 ± 0.27	11.09 ± 0.96	10.42 ± 0.27
Photometry (Megazyme set)	Total d-glucans *	54.78 ± 1.94	35.84 ± 0.83	30.07 ± 0.94
	*α*-d-Glucans *	9.99 ± 1.99	5.59 ± 0.63	5.72 ± 0.19
	*β*-d-Glucans *	44.79 ± 1.01	30.26 ± 0.44	24.35 ± 1.04
CITC-CZE	Chitin *	15.20 ± 2.27	2.92 ± 0.56	6.93 ± 0.56
	*N* (chitin)	0.93	0.18	0.43
OEA + CITC-CZE	*N* (proteins)	1.33	1.63	5.94
	Proteins *	9.94	12.06	43.63

* In dry matter.

## Data Availability

Data are contained within the article and supplementary materials.

## Supplementary Materials

The following supporting information can be downloaded at: https://www.mdpi.com/article/10.3390/jof10010023/s1, Table S1: Specification of the reference compounds; Figure S1: ATR-FTIR spectra of the reference compounds: (a) linseed oil, (b) wheat gluten, (c) chitin from crab shells, (d) potato starch, (e) fungal (1 → 3)-*α*-d-glucan, (f) yeast (1 → 3)(1 → 6)-*β*-d-glucan; Figure S2: ATR-FTIR spectra of ganoderic acids A, B, and D; Table S2: Infrared band assignment for the triterpenoid-rich surface layers of *Ganoderma* basidiocarps in comparison with the reference data for ganoderic acids A, B, and D.
