# Supplementary material for: Mid-Infrared Spectroscopic Study of Cultivating Medicinal Fungi Ganoderma: Composition, Development, and Strain Variability of Basidiocarps
Source: J Fungi (Basel). 2023 Dec 28;10(1):23. doi: 10.3390/jof10010023 (PMC10817577; doi:10.3390/jof10010023)
Supplement: Supplementary file 1 [file jof-10-00023-s001.zip › jof-2773738-supplementary.pdf]

# Supplementary material

**Table S1**

Specification of the reference compounds

| **Compound** | **Class** | **Source** | **Producer** | **FTIR** |
|---|---|---|---|---|
| Gluten | Proteins | Wheat grains (*Triticum* sp.) | Sigma-Aldrich (St. Louis, MO, USA) | KBr tablet |
| Chitin | Polysaccharides | Crab shells | Sigma-Aldrich (St. Louis, MO, USA) | KBr tablet |
| Starch | Polysaccharides | Potato tubers (*Solanum tuberosum* L.) | Sigma-Aldrich (St. Louis, MO, USA) | KBr tablet |
| (1 → 3)-*α*-D-glucan | Polysaccharides | Basidiocarps of *Pleurotus ostreatus* | Cultivated by Ing. Rudolf Ryzner (Kojátky, Czech Republic) | KBr tablet |
| (1 → 3)(1 → 6)-*β*-D-glucan | Polysaccharides | Baker's yeast (*Saccharomyces cerevisiae*) | Sigma-Aldrich (St. Louis, MO, USA) | KBr tablet |
| Seed oil | Fats | Linseed (*Linum usitatissimum*) | Chain store | ATR |
| Ganoderic acids A, B and D | Triterpenes | Basidiocarps of *Ganoderma* sp. | Sigma-Aldrich (St. Louis, MO, USA) | ATR |

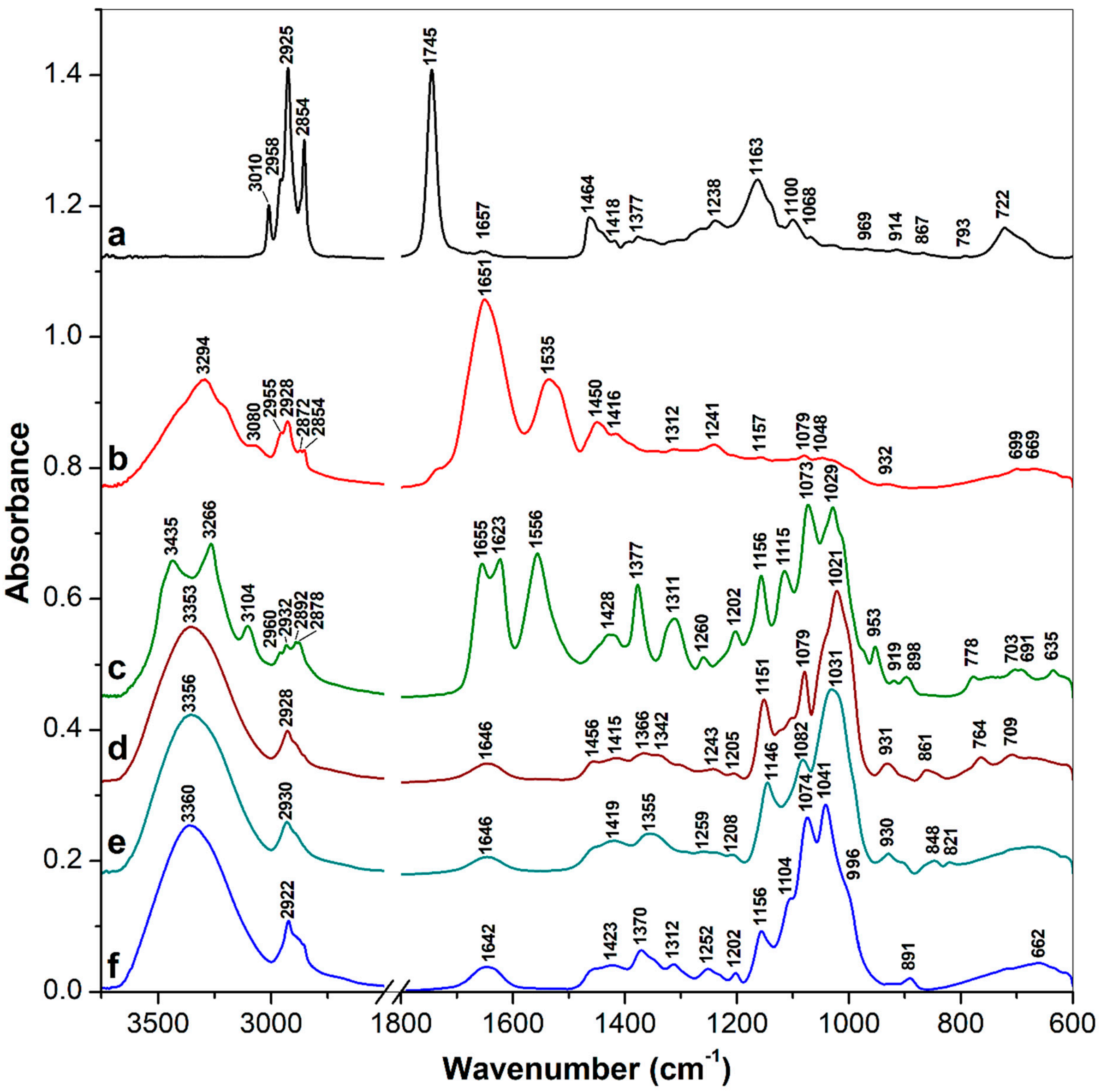


**Figure S1.** ATR-FTIR spectra of the reference compounds: (a) linseed oil, (b) wheat gluten, (c) chitin from crab shells, (d) corn starch, (e) fungal (1 → 3)-*α*-D-glucan, (f) yeast (1 → 3)(1 → 6)-*β*-D-glucan.

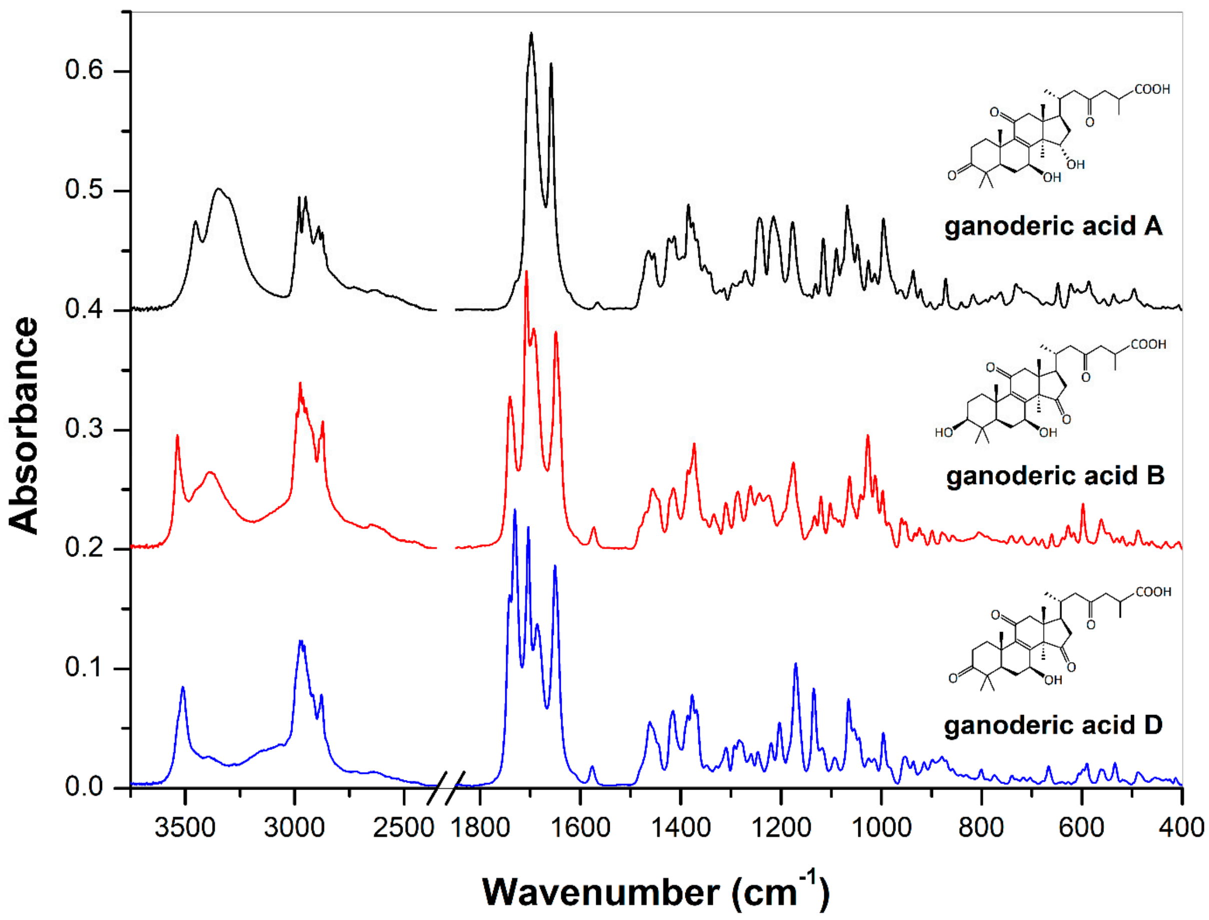


**Figure S2.** ATR-FTIR spectra of ganoderic acids A, B and D.

**Table S2**

Infrared band assignment for the triterpenoid-rich surface layers of *Ganoderma* basidiocarps in comparison with the reference data for ganoderic acids A, B and D [*]

| Sample | | | | | | | | | | | | | Standards | | | Band assignment |
|---|---|---|---|---|---|---|---|---|---|---|---|---|---|---|---|---|
| **1** | **4** | **5** | **6** | **7** | **8** | **9** | **10** | **11** | **12** | **13** | **14** | **15** | GA A | GA B | GA D | [53,54,65–69] |
| | | | | | | | | | | | | | 3452 | 3536 | 3511 | ν(OH) |
| 3301br | 3295br | 3356br | 3382br | 3373br | 3353br | 3332br | 3403br | 3394br | 3366br | 3380br | 3317br | 3303br | 3352br | 3389br | 3393br | ν(OH), ν($H_2O$) |
| 2979sh | 2965sh | 2971sh | 2970sh | 2978sh | 2967 | 2967sh | 2965 | 2976sh | 2972sh | 2967 | 2975sh | 2973sh | 2979 | 2974 | 2973 | $\nu_{as}(CH_3)$ |
| 2960sh | 2956 | 2951sh | 2965 | 2970sh | | 2956 | | 2958sh | 2957 | 2952sh | 2956sh | 2956 | 2950 | 2961 | 2967 | $\nu_{as}(CH_3)$ |
| 2935sh | 2934sh | 2929 | 2931 | | | 2939sh | 2935 | | | 2930 | 2931sh | 2936sh | | 2947 | 2956 | $\nu_{as}(CH_2)$ |
| 2924 | 2921 | 2919sh | 2920sh | 2929 | 2927 | 2921 | 2925sh | 2925 | 2925 | 2920sh | 2926 | 2925 | | 2926 | 2918 | $\nu_{as}(CH_2)$ |
| 2902sh | 2898sh | 2896sh | 2907sh | 2898sh | 2901sh | 2902sh | 2907sh | | | 2897sh | | 2903sh | 2890 | 2885 | | ν(CH) |
| 2877 | 2875 | 2880 | 2880 | 2872sh | 2878 | 2877 | 2880 | 2875 | 2877 | 2876 | 2873 | 2878 | 2874 | 2872 | 2877 | $\nu_s(CH_3)$ |
| 2854 | 2852 | 2850sh | 2851sh | 2852sh | 2855 | 2851 | 2850sh | 2856 | 2854 | 2851sh | 2854 | 2854 | 2860 | | | $\nu_s(CH_2)$ |
| 2648sh | 2662sh | 2638sh | 2660sh | 2647sh | 2670sh | 2652sh | 2656sh | 2657sh | 2678sh | 2648sh | 2630sh | 2655sh | 2631br | 2655br | 2639br | $\nu(OH)_{COOH}$ |
| 1748sh | 1749sh | 1743sh | 1743sh | 1750sh | 1749sh | 1741sh | 1751sh | 1747sh | 1742sh | 1740sh | 1749sh | 1747sh | | 1740 | 1741 | ν(C=O) (5-m. ring) |
| 1726sh | 1737sh | 1735sh | | 1737sh | 1731sh | 1727sh | 1735sh | | 1734sh | 1728sh | 1725sh | 1731sh | | | 1731 | ν(C=O) (5-m. ring) |
| 1703 | 1701 | 1699 | 1703 | 1703 | 1704 | 1703 | 1704 | 1704 | 1703 | 1704 | 1703 | 1702 | 1698 | 1708 | 1704 | ν(C=O) (open chain) |
| 1678sh | 1685sh | 1688sh | 1687 | 1687sh | 1680sh | | | | 1685sh | | 1682sh | 1682sh | | 1693 | 1686 | ν(C=O) (6-m. ring) |
| 1657 | 1655 | 1657 | 1659 | 1648sh | 1658 | 1657 | 1665 | 1660 | 1657 | 1658 | 1657 | 1658 | 1659 | 1649 | 1651 | ν(C=O) (α,β-unsat.) |
| 1580sh | | 1586 | 1585 | | 1581sh | 1579 | | 1583sh | | | 1578 | 1578sh | 1566 | 1573 | 1577 | ν(C=C) |
| 1472sh | 1469sh | 1471sh | 1471sh | 1469sh | | 1471sh | 1468sh | | 1467sh | | 1471sh | 1470sh | 1464 | 1470 | 1462 | $\delta_{as}(CH_3)$ (ang.) |
| 1460 | 1459 | 1456 | 1455 | 1457 | 1461 | 1461 | 1457 | 1460 | 1457 | 1462 | 1460 | 1460 | 1453 | 1456 | | $\delta_{as}(CH_3)$ (cycl.), $\delta(CH_2)$ |
| 1422sh | 1419sh | 1427sh | 1426sh | 1428 | 1426sh | 1423sh | | | 1429sh | 1424sh | 1423sh | 1426sh | 1424 | | | $\delta_{as}(CH_3)$ (open chain) |
| 1415 | 1415 | 1416sh | 1416 | 1413sh | 1411 | 1415 | 1415 | 1416 | 1412 | 1413 | 1417 | 1413 | 1413 | 1415 | 1416 | $\delta_{as}(CH_3)$ (cycl.), δ(OH) |
| 1388sh | 1386sh | 1385sh | 1386sh | 1386sh | 1387sh | 1388sh | | | 1387sh | 1387sh | 1388sh | 1388sh | 1385 | 1386 | 1386 | $\delta_s(CH_3)$ (ang. 5/6 m. rings) |

| | | | | | | | | | | | | | | | | |
|---|---|---|---|---|---|---|---|---|---|---|---|---|---|---|---|---|
| 1380 | 1378 | 1378 | 1373 | 1377 | 1380 | 1379 | 1377 | 1381 | 1377 | 1379 | 1380 | 1379 | 1376 | 1373 | 1377 | $\delta_s(CH_3)$ (ang. 6/6 m. rings) |
| 1364sh | 1363sh | 1362sh | | 1362sh | 1363sh | 1363sh | | 1363sh | 1362sh | 1364sh | 1364sh | 1363sh | 1368 | | 1368 | $\omega(CH_2)$, $\delta_s(CH_3)$ (cycl.) |
| 1346 | | 1346sh | 1344sh | 1348sh | 1346sh | 1344sh | 1342sh | 1344sh | 1346sh | 1344sh | 1345 | 1347sh | 1353 | 1351 | 1348 | $\delta_s(CH_3)$ (open chain) |
| | 1339sh | 1334sh | 1333sh | 1332sh | 1339sh | 1331sh | 1332sh | | 1332sh | 1334sh | | 1333sh | 1341 | 1334 | 1329 | δ(CH), δ(OH) |
| 1308sh | 1309sh | 1311 | 1311 | 1313 | 1314 | 1311sh | 1311sh | 1313sh | 1311sh | 1314sh | 1312sh | 1311sh | 1314 | 1309 | 1309 | δ(CH) |
| | | | | | | | | | | | | | 1297 | | 1293 | |
| | | 1284sh | | | | | | | | | | | 1281 | 1286 | 1283 | $\omega(CH_2)$ |
| 1274 | 1274 | | 1270 | 1271 | 1277 | 1277 | 1271sh | 1273 | 1273 | 1277 | 1275 | 1276 | 1271 | 1261 | 1259 | ν(CC), $\omega,\tau(CH_2)$, δ(CH) |
| 1244 | 1247sh | 1243 | 1247 | 1249 | 1244 | 1245 | 1245 | 1245sh | 1247sh | 1246 | 1245 | 1242 | 1244 | 1243 | 1246 | δ(OH), ν(CC), δ(CH) |
| 1228sh | 1226 | 1224sh | 1239sh | 1227sh | 1227 | | 1227sh | 1232 | 1230 | | 1227sh | 1228 | | 1225 | 1220 | $\nu(CO)_{acid, ester}$ |
| 1216sh | 1212sh | 1207 | 1207 | 1208 | 1217sh | 1220sh | 1207sh | 1207sh | 1208 | 1219 | 1211sh | 1217sh | 1215 | | 1203 | δ(OH), δ(CH), $\tau(CH_2)$ |
| 1172 | 1174 | 1166sh | 1167sh | 1180 | 1175 | 1173 | 1175 | 1174 | 1175 | 1174 | 1173 | 1173 | 1177 | 1175 | 1171 | ν(CC), δ(OH) |
| 1154sh | 1162sh | 1152sh | 1155sh | 1166sh | 1162sh | 1158sh | 1148 | 1145sh | 1149 | 1159sh | 1155sh | 1161sh | 1144 | | | ν(CC), $\tau(CH_2)$, δ(OH) |
| 1135 | 1136 | 1137sh | 1134sh | 1140sh | 1134 | 1132sh | | | 1129sh | 1134 | 1131sh | 1133 | 1133 | 1133 | 1135 | ν(CO), δ(OH), $\tau(CH_2)$ |
| 1113 | 1116 | 1115 | 1113 | 1114 | 1116 | 1114 | 1114 | 1113 | 1114 | 1115 | 1113 | 1115 | 1116 | 1120 | 1119 | ν(CC), ring vibrations |
| 1098 | | 1096 | 1095sh | 1099sh | 1098sh | 1101 | | 1097sh | 1100sh | 1097sh | 1099 | 1097sh | | 1102 | | ν(CC), ν(CO), δ(OH), δ(CH) |
| | 1089 | | | | 1086sh | | 1080sh | | | | | | 1090 | 1092 | 1093 | ν(CC), ν(CO), δ(OH), δ(CH) |
| | | 1077sh | 1077 | 1078sh | | | | | | | | 1086sh | | 1083 | | ν(CC), ν(CO), δ(OH), δ(CH) |
| 1063 | 1065sh | | | | | 1063 | 1062sh | 1063sh | 1063sh | 1063 | 1064 | | 1068 | 1064 | 1065 | ν(CO), ring breath |
| | | | 1049 | 1055 | 1047 | 1051 | | 1047 | 1049 | 1048 | 1052 | | | | 1054 | ν(CO), ring breath |
| 1044 | 1042 | 1046 | | | | | 1038 | | | | | 1045 | 1047 | 1041 | 1044 | ν(CO), ring breath |
| 1025sh | 1028sh | 1036 | 1035 | 1035 | 1025sh | 1025sh | | | | 1025sh | 1025sh | 1026sh | 1024 | 1027 | 1025 | $\omega(CCH_3)$ |
| 1014sh | 1012sh | 1018 | 1016 | 1016 | 1015sh | 1015sh | 1014sh | 1016sh | 1013sh | 1014sh | 1015sh | 1014sh | 1013 | 1012 | 1014 | $\omega(CCH_3)$, ring def. |
| 997 | 997 | 992sh | 993sh | 1002sh | 997 | 996 | 991sh | 995sh | 992sh | 996 | 997 | 997 | 996 | 997 | 996 | ring breath, δ(CH) |
| 975sh | 975sh | 977 | 975 | 984sh | 976sh | 975sh | 977sh | 976sh | 976sh | 975sh | 975sh | 975sh | | 985 | | ν(CC), δ(CH) |
| 957 | 956 | 958sh | 960sh | 960sh | 957 | 958 | 957sh | 956sh | 957sh | 958 | 957 | 958 | 963 | 959, 952 | 952 | ν(CC), ring vibrations |

| | | | | | | | | | | | | | | | | |
|---|---|---|---|---|---|---|---|---|---|---|---|---|---|---|---|---|
| 941 | 940 | 941 | 938 | 941 | 941 | 937sh | 930 | 939sh | 938sh | 941 | 939 | 941 | 936 | 933 | 936 | ν(CC), ω($CCH_3$) |
| 919 | 922 | 921 | 926 | 921 | 920 | | | | | | | 921 | 922 | 925 | | ν(CC), ρ($CH_2$) |
| | | | | | | 918 | 916sh | 918sh | 919sh | 919 | 918 | | | 917 | 915 | |
| 892 | 899 | 905 | 884 | 886 | 895 | 891 | 903sh | 895sh | 904 | 892 | 892 | 893 | 903 | 899 | 899 | ν(CC), ring def. |
| 876 | 876 | | | | 876 | 876 | | 881 | 874 | 875 | 876 | 875 | | 879 | 879 | ring vibr., ρ($CH_2$) |
| | | 868 | 868 | 867 | | | 869 | | | | | | 872 | | 871 | |
| | | | 846 | | | | 848 | 845sh | 850sh | | | | | 858 | 858 | ν(CC), ring def. |
| 842 | 841 | 843 | | 842 | 832 | 840 | | | | 838 | 840 | 836 | 841 | | 842, 831 | |
| 812 | 821 | 814 | 813 | 813 | 818 | 813 | 815 | 813 | 813 | 815 | 812 | 815 | 818 | 806 | 801 | ν(CC), ring def., ρ($CH_2$) |
| 793 | 791 | 800sh | 798sh | 798sh | 793 | 796 | 799 | 793 | 791 | 795 | 794 | 790 | 793 | 789 | 790 | ν(CC), ρ($CH_2$) |
| 773sh | 779 | 778 | 779 | 778 | 770sh | 776 | 769 | 769sh | 776sh | 769sh | 779 | 779 | 780 | 775 | 774 | ν(CC), ring def. |
| 742 | 743 | 744 | 742 | 742sh | 742 | 743 | 745sh | 748sh | 744sh | 744 | 743 | 743 | 762 | 756 | | open chain def. |
| 718 | 721 | 724sh | 717sh | 721sh | 719 | 720 | 721sh | 723sh | 719sh | 723 | 717 | 719 | 732 | 741 | 740 | ring and open chain def. |
| 693 | 709 | 712sh | 699sh | 714 | 706sh | 709sh | 713sh | 717 | 715 | 714 | | | 713 | 720 | 717 | ring def. |
| | 696 | 698sh | | 696sh | 695 | 695 | 700sh | 700sh | 695sh | 700 | 696 | 703sh | | 695 | 703 | ring vibr. |
| 682sh | 687 | 685sh | 683 | 681 | | | 687sh | 684sh | 671sh | | 684sh | 683 | | 680 | | ring vibr. |
| 668 | 673 | 677 | 669 | 661 | 668 | 669 | 669 | | 663sh | 666 | 667 | 671 | 670 | 661 | 666 | ring vibr. |
| 651 | 650 | 650 | 642sh | | 651 | 649 | | | 649sh | 651 | 650 | 652 | 648 | 638 | 639 | δ(CCC), δ(CCO) |

* GA, ganoderic acid; br, broad; sh, shoulder; ν, stretching; $_{as}$, antisymmetric; $_{s}$, symmetric; vibr., vibration, def., deformation; 5(6)-m., 5/6 m., five(six)-member; unsat., unsaturated; ang., angle; ang. 5/6 m., angle between the five-member and six-member rings; cycl., cyclic; δ, (in-plane) bending vibration; ω, waging vibration; τ, twisting vibration; ρ, rocking vibration.
